# A novel homozygous splice donor variant in the *LRPPRC* gene causing Leigh syndrome with epilepsy, a French-Canadian disorder in a Saudi family: case report

**DOI:** 10.3389/fped.2023.1288542

**Published:** 2023-11-17

**Authors:** Osama Y. Muthaffar, Angham Abdulrhman Abdulkareem, Abrar Ashi, Muhammad Imran Naseer

**Affiliations:** ^1^ Department of Pediatrics, Faculty of Medicine, King Abdulaziz University, Jeddah, Saudi Arabia; ^2^ Center of Excellence in Genomic Medicine Research, King Abdulaziz University, Jeddah, Saudi Arabia; ^3^ Department of Biochemistry, Faculty of Science, King Abdulaziz University, Jeddah, Saudi Arabia; ^4^ King Fahd Medical Research Center, King Abdulaziz University, Jeddah, Saudi Arabia; ^5^ Department of Medical Laboratory Sciences, Faculty of Applied Medical Sciences, King Abdulaziz University, Jeddah, Saudi Arabia

**Keywords:** WES, *LRPPRC*, Leigh syndrome, French-Canadian disorder, epilepsy, developmental delay, Saudi Arabia

## Abstract

**Background:**

The mitochondria are a cellular power house. Tissues are involved in frequent energy consumption, and any failure or irregularity in the continuous energy production could lead to abnormalities. The leucine-rich pentatricopeptide repeat (*LRPPRC*) gene is one of the mitochondrial-related functions genes; variations in these genes are responsible for complex phenotypes that affect many organs such as the brain, liver, and muscles.

**Materials and methods:**

This study enrolled a family with Leigh syndrome-like phenotype. The molecular diagnosis was conducted by first performing whole exome sequencing (WES), followed by Sanger sequencing.

**Results:**

A novel splice-site variant (c.469 + 2T > A) at the exon–intron boundary in the *LRPPRC* gene was identified using the WES data analysis. Sanger validation confirmed the autosomal recessive inheritance of the identified variant. Based on the ACMG criteria for variant classification, PVS1 and PM2 suggest that the identified variant in the *LRPPRC* gene is likely to be pathogenic.

**Conclusion:**

To the best of our knowledge, there have been no previous reports of this variant in the *LRPPRC* gene. Our research not only identifies a novel variant in the *LRPPRC* gene, but also confirms the unresolved molecular diagnosis of the family. WES can be used as a first-line diagnostic tool in familial cases, particularly in those cases when detailed clinical phenotyping is not possible. Once the molecular diagnosis is confirmed in a family, it is necessary to conduct a thorough re-evaluation of the patients’ specific clinical phenotypes in order to establish a clear genotype–phenotype correlation.

## Introduction

The genetic disorders may be caused by aberrations in chromosomal number or structure, as well as from point mutations occurring in a single gene, i.e., monogenic, or more than one gene, i.e., multigenic, or additional environmental factors, i.e., multifactorial. Monogenic disorders caused by alterations in a single gene follow Mendelian inheritance (1). Most of the monogenic diseases are caused by the point mutations, i.e., mutation altering single nucleotide, e.g., substitution, deletion, and insertion. Apart from chromosomal or nuclear DNA alterations, mitochondrial DNA variation also contributes to genetic diseases. These genes either contribute directly or in combination with the nuclear DNA gene expression. Abnormalities in oxidative phosphorylation pathways leading to abnormal musculoskeletal and neurodevelopmental disorders are a well-known example of mitochondria-related disorders (2).

Leigh syndrome, a severe neurological condition, typically manifests during the first year of life. Within 2–3 years, respiratory failure is the primary cause of mortality in individuals experiencing psychomotor regression, a condition characterized by a progressive loss of mental and physical functions. Only a small portion of people develop symptoms as adults or see their symptoms get worse with time. Infants with Leigh syndrome usually experience vomiting, diarrhea, and dysphagia as their initial signs of the disorder, which affects their ability to feed. These problems typically prevent a person from growing and gaining weight at the expected rate (failure to thrive). Severe muscular and movement problems are typical in Leigh syndrome. People who are affected may experience uncontrollable muscle spasms, hypotonia, dystonia, uncontrollable eye movements, and ophthalmoparesis and low muscle tone, as well as balance and mobility problems (ataxia). Peripheral neuropathy, a characteristic manifestation observed in patients diagnosed with Leigh syndrome, is associated with developing limb paralysis and loss of feeling, and can also cause movement issues.

Patients with Leigh syndrome develop brain tissue that resembles lesions, which contributes to the signs and symptoms of the illness. During a medical examination known as magnetic resonance imaging (MRI), distinctive lesions are discovered in particular regions of the brain. These include the brainstem, which connects the brain to the spinal cord and controls functions such as breathing and swallowing, the cerebellum, which governs balance and coordinates movement, and the basal ganglia, which help control movement. Brain lesions are commonly accompanied with demyelination, a condition that impairs the neurons’ capacity to activate the muscles that are used for movement or transmit sensory data from the rest of the body back to the brain. The mitochondria, serving as the primary energy-producing center of the cell, contribute approximately 90% of the total energy utilized by these cells.

There are numerous even thousands of mitochondria that use oxygen and convert food into energy. Similar to the nucleus, the mitochondria possess their own distinct genetic materials. A serious and long-lasting genetic malfunction can result from a DNA error in a mitochondrial gene. The mitochondrial genetic disease will start to manifest at birth or later in life at any age. Every organ in the body, including the brain, muscles, nerves, liver, kidneys, and heart, can be impacted by mitochondrial dysfunction. Approximately 1,000–4,000 children in the United States are born each year with mitochondrial disorder, which affects one in 5,000 persons (3).

Consanguinity, which is the union of close blood relatives, raises the possibility that a kid will inherit two copies of a deleterious gene. Consanguinity has a significant role in the development of genetic disorders, particularly in children who inherit genetic diseases as a result of the consanguinity (4).

The leucine-rich pentatricopeptide repeat (*LRPPRC*) gene, which has 4.8 kb and codes for a 130 kDa protein, was initially discovered in HepG2 cells in 1994 by Hou J. (5, 6). Subsequently, it was found that a decrease in *LRPPRC* level and steady-state levels of mitochondrial transcripts were responsible for the development of Leigh syndrome French-Canadian (LSFC). The causative role of the *LRPPRC* gene mutation in Leigh syndrome has been established over the previous few decades.

Congenital lactic acidosis is a characteristic of the genetically homogenous condition known as LSFC. The patients frequently exhibit neurological changes, developmental delays, abnormal facial features, and acute crises such as lactic acidosis, hyperglycemia, hepatic cytolysis, and hyperglycemia (7–10).

In addition, with the exception of LSFC, LRPPRC has the potential to be involved in early onset, multisystemic, and neurological manifestations of mitochondrial disorders (11, 12).

## Material and methods

### Ethical approval and biosafety measures

#### Sample collections and ethical approval

Ethical approval (013-CEGMR-02-ETH) was obtained from the local ethical committee of the Center of excellence in Genomic Medicine Research, King Abdulaziz University Jeddah. The sample collection and experimental procedures were conducted in accordance with the international guidelines outlined in the Declaration of Helsinki 2013. The blood samples of the whole family were collected for the purpose of this study, following the acquisition of informed written consent from the patient or their legal guardians, in the case of children participating in the study. In the center, the DNA extraction process as previously described (13, 14), involved utilizing the blood sample obtained. The Nanodrop™ 2000/2000c spectrophotometers (Thermo Fisher Scientific Waltham, MA, USA) were utilized.

#### Enrollment of the patients and family history

The index patient (IV-2) was born to a consanguineous couple residing in Saudi Arabia. The family exhibited a positive history of genetic disorders, with the elder brother of the affected child had ceased in early infantile age. The affected child had intrauterine developmental delays, and further brain MRI scans after birth revealed the absence of the carpus callosum. She was suspected of exhibiting neurodevelopmental disorder, specifically experiencing seizures. However, the specific clinical characteristics pertaining to the complete phenotype were not clear.

Peripheral blood samples from both the unaffected parents and the affected patient were collected in EDTA tubes. Genomic DNA extraction was performed, and a strategy for whole exome sequencing (WES) was designed for the molecular diagnostics of the patient. The variant identified in the exome sequencing data was cross-validated using Sanger sequencing in the family members who were available for Mendelian segregation analysis.

#### Whole exome sequencing

The whole exome sequencing of the index patient was performed using the collaborative facility of 3Billion Rare Diseases Diagnostics facility in South Korea. The data was generated using NOVASEQ6000 (Illumina, USA), and the prioritization of the genes was performed using an artificial intelligence-based analysis tool called EVIDENCE, developed by 3Billion, Republic of Korea. The data analysis also included the comparison of variants frequencies across various public databases such as gnomAD (http://gnomad.broadinstitute.org/) and ClinVar (https://www.ncbi.nlm.nih.gov/clinvar/). The clinical association with the phenotype was assessed using the human phenotype ontology (HPO) database (https://hpo.jax.org/app/). The pathogenicity of the identified variants was based on the guidelines of the American College of Medical Genetics 2015. The PVS1 criteria, as described in the aforementioned methods, are as follows: the null variant in a gene represents a genetic alteration where the loss of function is a known mechanism of the disease. This variant has an extremely low frequency in the genome Ad pop database, as indicated by the PM2 classification.

#### Sanger sequencing for variants validation

Sanger sequencing was conducted on the available family members to determine the identified variant in the *LRPPRC* gene. The process involved two steps: (1) the amplification of the target variant using the conventional PCR, and (2) the sequencing of the PCR by a chain termination reaction. The wild-type sequence of the *LRPPRC* gene was obtained from Ensembl genome browser using the genomic data map of version 37 (https://grch37.ensembl.org/index.html). The primer sequences were selected using the Primer3Plus online software (https://www.bioinformatics.nl/cgi-bin/primer3plus/primer3plus.cgi).

## Results

### Clinical history

**Figure 1:**
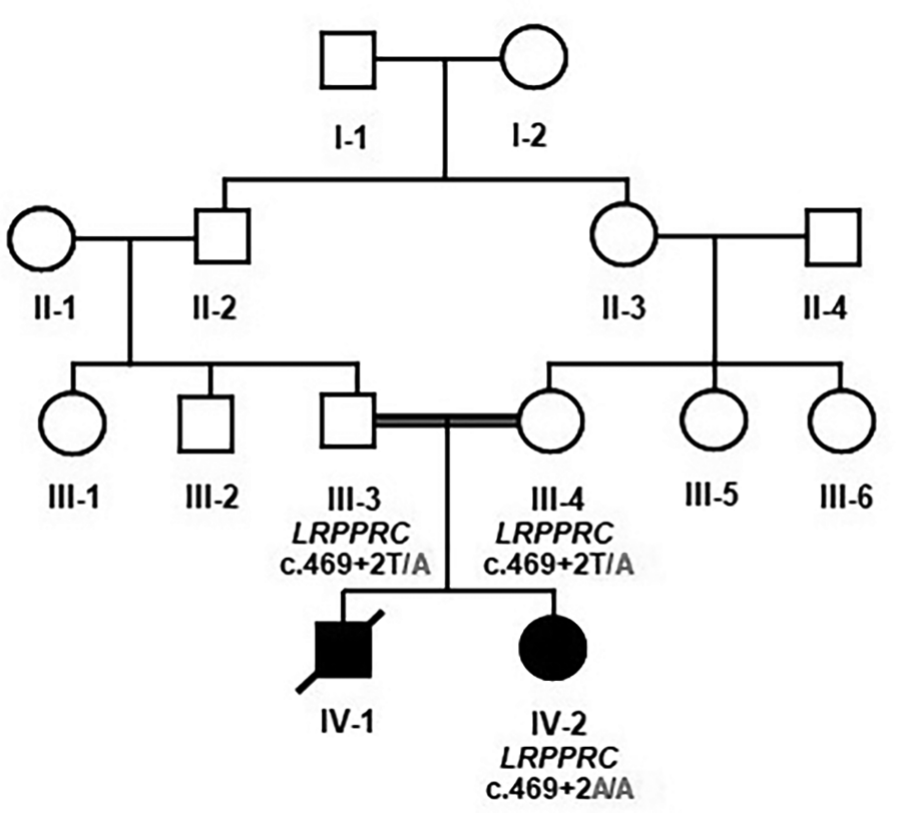
Pedigree analysis and Mendelian segregation of the identified variant. Unaffected father III-3 and unaffected mother III-4 were heterozygous T/A, and the affected daughter IV-2 was homozygous having A/A. The boxes denote male, and the circles denote female individuals.

The family had an affected female individual. The patient was born to a consanguineous union. It was a preterm delivery after a 34-week gestation. She had severe intrauterine growth retardation with an absent corpus callosum. She had persistent metabolic acidosis characterized by high levels of lactic acid. She exhibited a severe coarctation of the aortic arch as well as ambiguous genitalia. The family had a previous history of a similar male baby who had died in early infancy. He also had the lactic acidosis and intrauterine growth retardation. He had descendant testicles. The clinical diagnosis was consistent with either mitochondrial diseases or primary lactic acidosis. A detailed family pedigree was drawn subsequent to obtaining pertinent details from the parents as shown in Figure 1.

#### Genetic analysis

**Figure 2:**
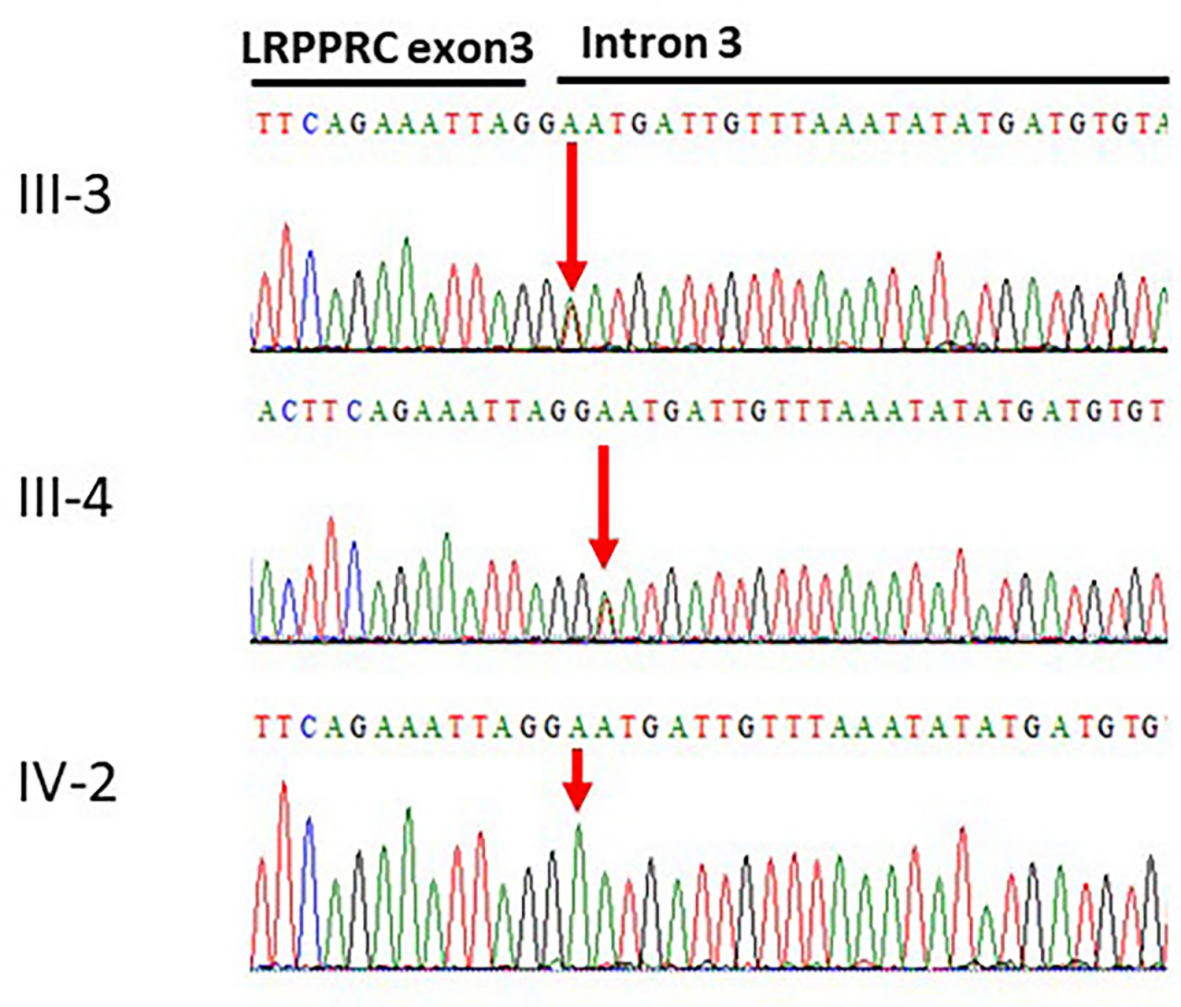
Sanger sequencing of the *LRPPRC* gene. The affected individual IV-2 has homozygous signal of the adenine nucleotide. The obligate carriers in the family father III-3 and mother III-4 had heterozygous signals for the thymine and adenine nucleotides c.469 + 2T > A.

The whole exome sequencing revealed a novel splice-site variant (c.469 + 2T > A) at the exon–intron boundary of the *LRPPRC* gene. The validity of this variant was confirmed using Sanger sequencing, which involved screening both parents. The two parents were heterozygous for the identified variant, while the patient carried the variant in a homozygous fashion. Thus, Sanger sequencing validated the autosomal recessive inheritance of the identified variation (Figure 2). To our knowledge, this variant has not been previously reported in the literature. Furthermore, the variant is novel in the gnomAD exomes and 1000 genomes.

The TraP score of the position 2-44206963-A-T was determined to be 0.903, indicating a potential detrimental effect. Furthermore, the variants obtained from sequencing were filtered to include only those that were novel/rare (MAF = 0.01%). These variants were then classified as functionally predicted damaging based on SIFT and Polyphen. The variant was also predicted as disease-causing by Mutation Taster. Moreover, the variant was not identified in either the ExAC or the 1000G databases.

Furthermore, we have conducted a literature review on the pathogenic variants identified in the *LRPPRC* gene, as shown in Table 1. All patients presented with poor acquisition of developmental milestones, including hypotonia. The patients exhibited global developmental delay across various domains, including gross motor skills, fine motor skills, language development, and cognitive abilities.

**Table 1:** List of pathogenic variations with available phenotype data known in the *LRPPRC* gene so far.

S. No.	c.DNA	Protein	Phenotype	Hypotonia *N* = 10/14 71.4%	Global developmental delay *N* = 11/14 78.5%	Cranial MRI *N* = 10/14 71.4%	Hypoglycemia *N* = 9/14 64.2%	References
1	c.1061C > T	p.Ala354Val	Cytochrome C oxidase deficiency/Leigh syndrome, FC	n.d	n.d	n.d	n.d	(7)
2	c.1178T > G	p.Tyr393Asp	Leigh syndrome, FC	n.d	Yes	Yes	n.d	(15)
3	c.1253A > C	p.Asn418Thr	mitochondrial DNA depletion syndrome	Yes	Yes	Yes	n.d	(16)
4	c.1792C > T	p.Gln598Ter	Intellectual disability/Mitochondrial disorder	Yes	Yes	n.d	Yes	(17)
5	[c.1921-7A > G:	Splicing:	Leigh syndrome, FC	Yes	Yes	Yes	Yes	(18)
	c.2056A > G] (CH)	p.Ile686Val						
6	c.2741C > A	p.Pro914Gln	Infantile mitochondrial disease, Lethal	Yes	Yes	Yes	Yes	(16)
7	[c.3130C > T c.3430C > T c.4078G > A] (CH)	p.Arg1044Ter p.Arg1144Cys p.Ala1360Thr	Leigh syndrome, FC	Yes	Yes	Yes	Yes	(12)
8	c.3809C > T	p.Ala1270Val	Complex I deficiency	Yes	Yes	Yes	Yes	(19)
9	c.469 + 1G > A	Splicing	Leigh syndrome, FC	n.d	n.d	n.d	n.d	(20)
10	c.1155 + 30A > G							
	c.3570-3C > T c.3900 + 14C > T c.3900 + 15C > T	Splicing	Parkinson's disease	n.d	n.d	n.d	n.d	(21)
11	c.1582 + 7A > G	Splicing	Cytochrome c oxidase deficiency	Yes	Yes	Yes	Yes	(11)
	c.3900 + 1G > T	p.(Gly1050Argfs*4)		Yes	Yes	Yes	Yes	
	[c.1582 + 7A > G	p. Glu497*		Yes	Yes	Yes	Yes	
	c.3147dupA] (CH)	p.Gly1050Argfs*4						
12	c.469 + 2T > A	Splicing	Infantile mitochondrial disease, Lethal	Yes	Yes	Yes	Yes	Present study

FC, French-Canadian type; n.d., not determined; CH, compound heterozygous.

Four patients data in the case of hypotonia, three patients data in the case of Global Developmental, four patients data in the case of cranial MRI, and five patient data in hypoglycemia were not determined (n.d).

Moreover, we also reported the *LRPPRC* variations enlisted in the gnomAD database with their predicted consequences. This information will provide the reader with updated insights into the understanding of genetic diseases. The variant classification criteria established by the ACMG, namely PVS1 and PM2, suggest that the identified variant in the *LRPPRC* gene is likely pathogenic. The segregation of the variant also supports its deleterious characteristics.

#### Sanger sequencing validation

The results from the Sanger sequencing analysis confirmed the presence of a novel splice-site variant (c.469 + 2T > A) at the exon–intron boundary in *LRPPRC* gene causing Leigh Syndrome with epilepsy, a French-Canadian disorder in a Saudi family.

## Discussion

The mitochondrion serves as a site for the production of cellular energy. The majority of the organs, including the muscles, brain, and lungs, frequently acquire energy through the process of cellular respiration occurring within the mitochondria. The genes that are responsible for mitochondrial functions are found on both the mitochondrial and chromosomal DNA. Any abnormality in the normal functions of the mitochondrial cascade has the potential to abnormal functions. The current demand in familial mitochondrial disorders necessitates the implementation of molecular diagnosis (22). The diagnosis of patients with incomplete phenotypic expression poses challenges in molecular biology laboratories (23). However, the recent advancements in next-generation sequencing have brought about a significant transformation in the field of molecular diagnosis (24). This study demonstrated an association between a novel homozygous *LRPPRC* gene variant and a lethal type of infantile mitochondrial disease. Due to the patient’s limited survival time, a detailed clinical diagnosis was not possible.

The *LRPPRC* gene is located on the reverse strand of chromosome 2 (from genomic DNA position 43,886,224 to 43,996,226). The wild-type sequence of the *LRPPRC* gene can be retrieved using the accession number ENSG00000138095 from Ensembl Genome Browser (https://asia.ensembl.org/Homo_sapiens/Gene/). The gene consists of 38 exons coding for an mRNA molecule that is 6,603 bases in length (https://www.ncbi.nlm.nih.gov/nuccore/NM_133259.4), which in turn translates into a protein consisting of 1,394 amino acids.

The function of the *LRPPRC* has been reported in the assembly of cytochrome oxidase. Associations have been identified between *LRPPRC* variations and lower expression levels of mRNA for cytochrome c oxidase I and cytochrome c oxidase III. Such patients presented Leigh syndrome French-Canadian type phenotype, and they shared a founder variation (p.Aal354Val) in the LRPPRC protein (8).

The human gene mutations database has enlisted a total of 37 variations. The missense variations develop diverse phenotypes including premature ovarian insufficiency (25), tetralogy of Fallot (26), mitochondrial respiratory chain complex IV deficiency (27), Leigh syndrome, French-Canadian type (28), cytochrome c oxidase deficiency (9), infantile mitochondrial disease, lethal type (16), congenital heart disease (29), autism (30), inborn errors of metabolism (31), failure to thrive, microcephaly, global developmental delay, intellectual disability, hypotonia, and seizures (25). There are only two nonsense variations that result in developing Leigh syndrome French-Canadian type and mitochondrial disorder with intellectual disability (17, 28).

Splicing variations can also result in diverse phenotypes, such as Leigh syndrome, French-Canadian type (20), cytochrome c oxidase deficiency (11), inborn error of metabolism (31), and developmental disorder (30).

The splice-variant c.469 + 2T > A, which was identified in this study, was novel and had not been reported before. The previously reported variant c.469 + 1G > T was found in close proximity to the variant identified in our family. It was confirmed through laboratory analysis using whole genome sequencing. It had been clinically diagnosed as Leigh syndrome with French-Canadian type (20). The patient included in this present study also exhibited a limited life expectancy, similar to those previously reported for the c.469 + 2T > A variant.

## Conclusion

The utilization of whole exome sequencing has been adopted as a first-line molecular diagnostic tool in most of clinical setups. The phenotype developed by the *LRPPRC* gene is clinically diverse, and the survival rate in majority of cases is limited. Thus, relying solely on clinical features is insufficient for accurately determining a comprehensive diagnosis. Instead, it is more advantageous to perform whole exome sequencing in patients presenting with symptoms such as failure to thrive, developmental delays, signs of inborn metabolic disorders, intellectual disability, or suspected phenotype of mitochondrial disorders.

## Data Availability

The datasets for this article are not publicly available because family consent to publicly share the data was not allowed. Requests to access the datasets should be directed to mimrannaseer@yahoo.com.
